# Reduced organizational skills in adults with ADHD are due to deficits in persistence, not in strategies

**DOI:** 10.7717/peerj.9844

**Published:** 2020-09-08

**Authors:** Guillaume Durand, Ioana-Smarandita Arbone, Monica Wharton

**Affiliations:** 1 School of Counselling, Psychotherapy, and Spirituality, St. Paul University, Ottawa, Ontario, Canada; 2 Department of Public Health, Faculty of Medicine, University of Toronto, Toronto, Ontario, Canada; 3 School of Psychology, University of Ottawa, Ottawa, Ontario, Canada

**Keywords:** ADHD, Organizational skills, Gender differences, Academic achievements

## Abstract

**Background:**

Attention Deficit Hyperactivity Disorder (ADHD) is commonly associated with poor organizational skills, as per the definition of ADHD. However, the contribution of each of the following factors (and their interactions) to the aforementioned poor organization, while comparing individuals with and without ADHD, has not been analyzed in-depth: Work Organization; Communication Clarity; Punctuality; Goal-oriented behaviour; Assiduity; Workspace Organization; Strategies; and Attentiveness. The purpose is to examine the self-reported levels of organizational skills in adults with and without ADHD.

**Methods:**

Seven hundred seventy-four (*n* = 303 with a diagnosis of ADHD) adults from the community were recruited online. Participants completed a measure of organizational skills and a screening measure of ADHD.

**Results:**

Participants with a diagnosis of ADHD displayed lower scores than individuals without a diagnosis of ADHD on all organizational skills with the exception of using strategies to remain organized and learn new information. Higher levels of education were associated with higher levels of organizational skills. While there was no difference between males and females with ADHD, females without ADHD scored higher than males without ADHD.

**Conclusion:**

This study supports that individuals with ADHD can efficiently develop strategies, but may have difficulties using those strategies in a continuous manner. Suggestions to increase organizational skills in adults with ADHD are presented.

## Introduction

### Cognition and organizational skills

The term ‘organizational skills’ refers to a functional set of cognitive abilities which facilitate goal-oriented behaviour (Clark, Prior & Kinsella, 2002). Previous research has identified working memory as a significant causal mechanism for the execution of organizational skills by conceptualizing working memory as both a domain-central executive and general executive function (Kofler et al., 2011; Kofler et al., 2018). Despite its multiple conceptualizations, working memory generally refers to the cognitive process of temporary phonological and visuospatial information retention alongside simultaneous processing that is necessary for advanced cognition such as information encoding, learning, reason, and language (Baddeley, 1992). In this way, organizational skills are impacted by the functional capabilities of the working memory.

### Organizational skills in ADHD: cognitive and alternative explanations

Attention Deficit Hyperactivity Disorder (ADHD) is a neurodevelopmental disorder characterized by chronic inattention, hyperactivity, and impulsivity, the severity of which determines the individual’s ADHD presentation as either inattentive presentation (ADHD-IA), hyperactive-impulsive presentation (ADHD-HI), or combined presentation (ADHD-C) (American Psychiatric Association, 2013). In previous research, ADHD in adolescents was positively associated with the exhibition of anxiety and depression, the symptoms of which are linked to the impairment of executive functions (Molitor et al., 2019). Although there is relative scientific consensus regarding the phenotypic behaviours of ADHD, discrepancies still exist regarding their nature as either core features or as symptoms caused by neurocognitive impairments (Kofler et al., 2011).

According to the functional working memory model of ADHD, impaired working memory is a core feature of ADHD, while inattention, hyperactivity, and impulsivity are secondary features (Kofler et al., 2011). In this model, working memory deficits, directly and indirectly, affect behavioural outcomes, organizational skills, social skills, and cognitive performance (Kofler et al., 2011). Congruent with this model, previous research has yielded consistent evidence that suggests impaired working memory is characteristic of ADHD and has a functional relationship with ADHD-typical hyperactive and inattentive behaviours (Kofler et al., 2010; Rapport et al., 2009). In this context, the connection between organizational skills and ADHD is more apparent, as impaired working memory would inhibit the capacity to intake, integrate, and apply external information in ways necessary for goal-oriented behaviour such as organizational skills (Kofler et al., 2018).

In addition, some research has shown positive relationships between the occurrence of hyperactive behaviours and the cognitive demand of a task, which suggests that hyperactive motor engagement may serve as a functional compensation for ADHD’s deficits in the executive functions necessary for organizational behaviour (Kofler et al., 2018). Explanatory hypotheses largely identify this relationship as a compensatory function for working memory deficits; although, the proposed specificities of the process vary between dopaminergic cortical stimulation triggered by physiological arousal (Kofler et al., 2018; Rapport et al., 2009), motor activated metabolic functions that regulate serotonin synthesis by enhancing pre-serotonin metabolite transportation (Kofler et al., 2018; Patrick & Ames, 2015), and the externalization of working memory through external vocalizations which engage more efficient information processing through sensory encoding and the automatic phonological loop (Kofler et al., 2018). Regardless of the working hypothesis being applied, this positive relationship suggests functional reversibility of the influential relationship between working memory and ADHD typical behaviours proposed by the functional working memory model of ADHD.

Alternatively, the multiple-pathway model of ADHD identifies motivation/delay aversion as an additional factor which works alongside executive function deficits—such as working memory deficits—in contributing to the exhibition of behaviours and impairments symptomatic of ADHD (Molitor et al., 2019). In this model, consistently engaging in organizational skill behaviours requires motivation and immediate stimuli to reinforce the behaviours; however, in the absence of immediate reinforcement, the motivation to employ organizational skills is diminished and replaced with the motivation for other behaviours that will provide immediate stimulus reinforcement (Molitor et al., 2019). One implication of this model is that low motivation to perform could be the cause of ADHD’s difficulty employing working memory-based organizational skills, rather than a lack of knowledge (Molitor et al., 2019).

Therefore, working memory function is an important consideration for organizational skills in general and particularly for those with ADHD. In addition to cognitive factors, motivation and neurobiology also impact organizational skills in this population. The literature therefore suggests that organizational skills are less developed overall in those with ADHD, with no specification as to what organizational skills are less developed.

### Consequences of ADHD’s impaired organizational skills

Equally important to the functional intricacies of ADHD are the ways in which they impact personal success in adolescence and adulthood. In previous research, measuring the ability to implement organizational skills for time, task, and material management surpassed intelligence as an accurate predictor of future grade point average (GPA), demonstrating a negative relationship between organizational difficulty and academic success (Langberg et al., 2013). ADHD itself was also found to negatively affect long-term academic achievement (Arnold et al., 2020). There is also evidence that pharmacologic treatment improves core features of ADHD and academic productivity (Arnold et al., 2020; Loe & Feldman, 2007), though these treatments may not affect ultimate academic achievement (Loe & Feldman, 2007). Therefore, ADHD impacts academic achievement and lack of organizational skills negatively impacts this achievement. However, the relationship between ADHD and educational achievement—especially the role of organizational skills—is not yet clear.

Likewise, impaired organizational skills in ADHD produce behaviours detrimental to success in both finances and occupation by manifesting as habitual forgetfulness, tardiness, failure to complete tasks, failure to follow instructions, and a lack of adaptability (Langberg et al., 2013). In addition to financial and occupational problems, these behaviours also cause difficulties in creating and maintaining social relationships, often leading to self-destructive deviant behaviours such as substance abuse (Kofler et al., 2011).

### ADHD and gender

There is evidence that men are diagnosed with ADHD more frequently than women (e.g., Danielson et al., 2018). However, the reason for this discrepancy is not clear. Some research provides support for a potential female protective effect against ADHD behaviours, according to which females are more resistant to genetic and environmental factors that cause ADHD and therefore need more exposure to these factors to present the condition (Taylor et al., 2016). Other research discusses potential gender biases (Eryılmaz, 2019; Rollins, 2005), while other research discusses differences between men and women with respect to ADHD (Mowlem et al., 2019). For example, there may be cognitive differences between men and women that affect ADHD presentation (Skogli et al., 2013) as well as neurobiological gender differences (Onnink et al., 2014).

Because of these postulated gender differences, some research suggests that targeted coping strategies to manage ADHD symptoms—such as lack of organizational skills—may be particularly helpful for women with ADHD while men may benefit more from affiliating with prosocial peers (Elkins et al., 2018). While gender seems to impact ADHD and its core symptoms (e.g., organizational skills), the nature of this impact is not clear based on the current literature.

### Current study

The present study investigates organizational skills deficiencies commonly reported in individuals with ADHD. More specifically, we are interested in determining if individuals diagnosed with ADHD report lower scores on all organizational skills or only a select few. We hypothesize that adults with a diagnosis of ADHD will report lower scores on all organizational skills. We also hypothesize that individuals with higher levels of education will report higher levels of organizational skills. Finally, we will explore gender differences (that we hypothesize to be an important factor to be explored in future research), as well as the relative contribution of organizational skills on mean score of a self-reported measure of ADHD.

## Methods

### Participants

A total of seven hundred seventy-four (*N* = 774) participants were recruited online on reddit/r/SampleSize and Facebook groups for individuals with ADHD. Participants were 31% males and 69% females. A total of 303 participants (39% of the sample) reported having received an official diagnosis of ADHD from a physician, a psychiatrist, or a psychologist. Most participants were native English speakers (80%). Most participants had high school or college education (44%), followed by bachelor education (34%) and post-bachelor education (22%). Almost half (44%) of the participants reported currently being enrolled as a student in a university. Most participants were located in North America (64%) or Europe (27%). The mean age was 27.02 years old (*SD* = 8.38). All participants provided informed consent prior beginning the online study. Participants were not compensated for completing the present study. The study was approved and given ‘exempt’ status by the IntegReview Ethical Review Board (Austin, TX, USA), under protocol number 11022016.

### Measures

#### Durand organizational skills questionnaire (DOSQ; Durand, 2020)

The DOSQ is a 38-item self-reported questionnaire measuring one’s level of organizational skills through a total score and eight factors: Work Organization (i.e., organizing work-related supplies in an efficient manner and keeping track of the location of those supplies), Communication Clarity (i.e., communicating with others in a clear manner while avoiding misunderstandings), Punctuality (i.e., being punctual, arriving on time, and completing projects by their deadlines), Goal-oriented behaviour (i.e., having multiple specific goals and objectives in life, personally and professionally, and visualizing future objectives), Assiduity (i.e., working in a consistent manner, following and managing schedules, and staying focused on long-term projects), Workspace Organization (i.e., keeping a workspace organized for a long period of time after organizing it and to perform better in organized environments), Strategies (i.e., using strategies to keep track of schedules and deadlines and to use tools and systems when required to learn new information), and Attentiveness (i.e., being attentive, remembering details, and use efficient strategies to help remember information). Each item is rated on a 6-point rating scale ranging from *Strongly Disagree* to *Strongly Agree*.

During its development, the initial version of the DOSQ contained 200 items, whereas half were related to organization at work and the other half reflecting organization in life outside work. Item reduction was first performed by having a panel of experts rate the relevance of each item to organizational skills, which reduced the number of items to 87. Subsequently, a series of exploratory factor analysis (EFA; principal axis factoring with direct oblimin rotation) were conducted on all items of the DOSQ. Items were kept when loading .3 or higher on their target factor without loading .3 or higher on any other factor. A 9-factor solution emerged accounting for 66.46% of the variance. After inspection of intercorrelation of each scale to the total score, one factor was dropped, leaving the total to 38-item.

Subsequent to its development, the DOSQ’s psychometric properties were examined in two follow-up studies (Durand, 2020). In the first study (*N* = 542), a confirmatory factor analysis (CFA) supported the 8-factor structure of the DOSQ. The fit statistics were as follows: *χ*^2^ = 1459.516, *df* = 616, *χ*^2^/ *df* = 2.369, NFI = 0.87, NNFI = 0.91, CFI = 0.92, SRMR = 0.063, RMSEA = 0.050. Out of the 542 participants, a total of 262 also completed the Satisfaction with Life Scale (SWLS; Diener etal, 1985) and the State Self-Esteem Scale (SSES; Heatherton & Polivy, 1991). The DOSQ showed a weak positive association with SWLS (*r* = .28) and SSES (*r* = .25). A second study examined the association between the DOSQ and the Flourishing Scale (FS; Diener et al., 2010), the Hope Scale (THS; Snyder et al., 1991), and the Rational—Experiential Inventory (REI; Pacini & Epstein, 1999). The DOSQ showed a moderate association with FS (*r* = .43) and THS (*r* = .47), as well as a weak association with rational ability (*r* = .28). Overall, while additional studies focusing on the convergent and divergent validity of the DOSQ are needed, these results support the psychometric properties and the utility of the DOSQ as a measure of organizational abilities, as observed by its relationship with analytical thinking, focusing and planning to meet goals, and an overall feeling of competence and positivity.

#### Adult ADHD self-report scale (ASRS; Kessler et al., 2005)

The ASRS is a DSM-IV-based measure including 18 questions related to the frequency of the DSM-IV Criterion A symptoms of adult ADHD from the past six months. This self-report is rated on a 5-point scale, ranging from Never to Very Often. In addition to a screening score consisting of the total of the first six items, the ASRS provides a score on two subscales, namely Inattention and Hyperactivity-Impulsivity. The ASRS has demonstrated good psychometric properties across numerous studies (Kessler et al., 2007; Kim, Lee & Joung, 2013).

## Results

All analyses were performed by condition (no-ADHD/ADHD). Examination of Kurtosis and Skewness support a normal distribution on both the DOSQ and the ASRS, as well as their respective subscales. Table 1 shows the descriptive data (mean, standard deviation, range, and Cronbach’s alpha) for both conditions, as well as the Cohen’s *d* effect size on each scale between condition. A series of analysis of variance (ANOVA) supported mean differences between conditions on all scales at the exception of DOSQ-Strategies. The ANOVA results are as follow: DOSQ Total (*F* (1, 772) = 106.760, *p* < .001), Work Organization (*F* (1, 772) = 45.391, *p* < .001), Communication Clarity (*F* (1, 772) = 49.304, *p* < .001), Punctuality (*F* (1, 772) = 51.557, *p* < .001), Goal-Oriented behaviour (*F* (1, 772) = 8.807, *p* = .003), Assiduity (*F* (1, 772) = 190.181, *p* < .001), Workspace Organization (*F* (1, 772) = 9.076, *p* = .003), Attentiveness (*F* (1, 772) = 217.864, *p* < .001), ASRS screening (*F* (1, 772) = 251.877, *p* < .001), Inattention (*F* (1, 772) = 294.498, *p* < .001), and Hyperactivity Impulsivity (*F* (1, 772) = 168.528, *p* < .001).

**Table 1:** Descriptive data.

	No ADHD diagnosis (*n*= 471)			ADHD diagnosis (*n*= 303)			
	Mean (*SD*)	Range	*α*	Mean (*SD*)	Range	*α*	*d*
DOSQ							
Total^**^	136.36 (32.14)	44–208	0.94	114.55 (22.18)	52–186	0.86	0.79
Work Organization^**^	11.52 (3.50)	3–18	0.70	9.83 (3.32)	3–18	0.60	0.50
Communication Clarity^**^	22.83 (7.64)	6–36	0.94	19.00 (7.01)	6–36	0.93	0.52
Punctuality^**^	12.51 (4.44)	3–18	0.95	10.09 (4.81)	3–18	0.95	0.52
Goal–Oriented Behaviour^*^	13.20 (5.15)	4–24	0.85	12.11 (4.79)	4–24	0.81	0.22
Assiduity^**^	18.71 (6.78)	6–36	0.84	12.64 (4.44)	6–30	0.71	1.06
Workspace Organization^*^	19.21 (5.44)	5–30	0.84	18.04 (5.00)	5–30	0.78	0.22
Strategies	23.50 (8.27)	7–42	0.86	22.98 (7.93)	7–41	0.83	0.06
Attentiveness^**^	14.86 (5.01)	4–24	0.82	9.87 (3.84)	4–23	0.69	1.12
ASRS						
Screener^**^	18.60 (4.09)	9–29	0.64	23.13 (3.53)	12–30	0.59	1.19
Inattention^**^	28.10 (6.65)	10–45	0.84	35.81 (5.11)	20–45	0.76	1.30
Hyperactivity-Impulsivity^**^	24.57 (6.08)	10–44	0.78	30.46 (6.27)	11–45	0.81	0.95

**Notes.**

* *p* < .01 ** *p* < .001 indicates a significant diagnosis difference

Organizational skills were examined between and within conditions. Table 2 reports a series of ANOVA on education and gender between conditions. Overall, participants with no ADHD diagnosis scored higher on the DOSQ independently of their level of education or gender. Results regarding the DOSQ Total also emerged within groups. Within participants with no ADHD, higher levels of education was associated with higher levels of organizational skills (*F* (2, 468) = 17.575, *p* < .001). More specifically, participants in the post-bachelor category scored higher than those in the high school and college category (*p* < .001) as well as higher than those in the bachelor level category (*p* < .001). Within participants with ADHD, higher levels of education was also reflecting higher levels of organizational skills (*F* (2, 295) = 8.050, *p* < .001). Participants in the bachelor level category (*p* = .005), as well as those in the post-bachelor level category (*p* < .001) scored higher than participants in the high school and college category. For gender, while there was no difference between males and females with an ADHD diagnosis on the DOSQ, a difference was observed in the no-ADHD category (*F* (1, 469) = 4.916, *p* = .027), whereas females scored higher than males. To examine potential interactions, a Univariate ANOVA with DOSQ Total as dependent variable and education, gender, and ADHD diagnosis as fixed factors was performed. There was no significant interaction between education and gender (*F* (2, 753) = 0.676, *p* = .509), between education and ADHD diagnosis (*F* (2, 753) = 0.857, *p* = .425), between gender and ADHD diagnosis (*F* (1, 753) = 3.050, *p* = .081) and between education, gender, and ADHD diagnosis (*F* (2, 753) = 1.641, *p* = .194).

**Table 2:** DOSQ Total score by education and gender.

	No ADHD diagnosis		ADHD diagnosis				
	Mean (*SD*)	*N*	Mean (*SD*)	*N*	*F*	*p*	*d*	
Education							
High school / college	129.89 (31.06)	206	109.43 (20.77)	130	43.963	<.001	0.77		
Bachelor level	134.83 (31.06)	155	117.41 (22.46)	103	24.034	<.001	0.64		
Post-bachelor level	151.54 (30.97)	108	121.73 (21.60)	63	45.427	<.001	1.12		
Gender										
Males	132.01 (31.85)	170	114.69 (20.62)	72	18.042	<.001	0.64		
Females	138.81 (32.09)	301	114.51 (22.68)	231	95.785	<.001	0.87		

As shown in Table 3, a series of regression analyses were performed to examine the strength of each organizational skill in predicting ADHD symptoms. The first model examined the eight factors of the DOSQ, as well as education, against ASRS screening, which resulted in a significant model (*F* (8, 765) = 147.233, *p* < .001, *R^2^* = .606, *adjusted R*^2^ = .602) predicting 60.2% of the variance. Only Communication Clarity, Assiduity, and Attentiveness were significant predictors. The second model examined the DOSQ factors and education against ASRS Inattention, which resulted in a significant model (*F* (8, 765) = 300.113, *p* < .001, *R^2^* = .758, *adjusted R*^2^ = .756) predicting 75.6% of the variance. Only Communication Clarity, Assiduity, and Attentiveness were significant predictors. The third model examined the DOSQ factors and education against ASRS Hyperactivity-Impulsivity, which resulted in a significant model (*F* (8, 765) = 42.382, *p* < .001, *R^2^* = .307, *adjusted R*^2^ = .300), predicting 30.0% of the variance. Only Communication Clarity, Goal-Oriented behaviour, Assiduity, Strategies, and Attentiveness were significant predictors.

**Table 3:** Results of stepwise linear regression for ASRS.

	*B*	*t*	*p*
	ASRS Screening		
Constant		63.649	<.001
Communication Clarity	−0.119	−4.509	<.001
Assiduity	−0.428	−12.121	<.001
Attentiveness	−0.373	−11.217	<.001
	Inattention		
Constant		82.189	<.001
Communication Clarity	−0.151	−7.331	<.001
Assiduity	−0.430	−15.537	<.001
Attentiveness	−0.415	−15.921	<.001
	Hyperactivity-Impulsivity		
Constant		35.891	<.001
Communication Clarity	−0.196	−5.606	<.001
Goal-Oriented Behaviour	0.084	2.346	=.019
Assiduity	−0.199	−4.253	<.001
Strategies	0.117	3.08	=.002
Attentiveness	−0.374	−8.475	<.001

## Discussion

A proper understanding of ADHD and its relationship to organizational skills is necessary to devise proper interventions for the ADHD population. Indeed, the current findings support the initial hypotheses that (1) organizational skills differ between ADHD and non-ADHD groups; (2) educational attainment is related to better organizational skills, and (3) that organizational skills are affected by gender (though the precise relationship remains to be clarified in future research).

Overall, both questionnaires (ASRS screener and DOSQ) differentiate between ADHD and non-ADHD groups. Even when looking at the questionnaires’ subcomponents, ADHD and non-ADHD groups are clearly differentiated. The only exception to this broad differentiation is the DOSQ’s *Strategies* subscale, which shows that both groups are more or less equally able to develop organizational strategies. Upon further investigation of the subcomponents, the ASRS screener and DOSQ show the following relationships: a higher score on the *inattention* subscale (from the ASRS screener) is associated with lower levels on both DOSQ’s *Assiduity* and DOSQ’s *Attentiveness* scores, and to a lesser extent, on DOSQ’s *Communication Clarity*. Also, a higher score on the *hyperactivity-impulsivity* subscale (from the ASRS screener) is related to lower levels on the DOSQ’s *Attentiveness, Communication Clarity,* and *Assiduity*. However, a higher score on the *hyperactivity-impulsivity* subscale (from the ASRS screener) is also associated with higher levels on *Strategies* and *Goal-Oriented behaviour*.

This pattern of results is consistent with the hypothesis according to which ADHD causes executive functioning deficits (where executive functioning is defined as the arrangement of one’s behaviour and thinking in a manner that will lead to the desired result) rather than deficits in overall problem-solving (i.e., developing strategies) (Barkley, 2012). Furthermore, the connection between DOSQ’s *Assiduity* and ASRS’ *hyperactivity-impulsivity* matches previous findings suggesting that hyperactive symptoms may act to enhance one’s executive functioning (e.g., attention, overall arousal) when engaged in organizational-type activities (Kofler et al., 2019). While hyperactivity (through the mechanism of motor engagement) has been identified as a potential enhancer of general executive functioning (Kofler et al., 2018), this study clarifies that it is specifically *assiduity* that is particularly enhanced by such hyperactivity-impulsivity symptoms.

In addition, organizational skills relate to higher educational attainment, suggesting that those with ADHD may have more difficulty attaining higher education, particularly at the post-bachelor level. It is also possible that organizational skills may even be necessary to attain higher education. Therefore, interventions targeting organizational skills in those with ADHD are likely to be very beneficial in a higher education setting. Since DOSQ’s subscales of *Communication Clarity, Assiduity* and *Attentiveness* are the primary predictors of ADHD, targeting these areas is likely to be most beneficial.

However, the pattern of results (described thus far) should factor in gender.. Mirroring previous research, the present findings suggest that men and women with ADHD experience different challenges (Doidge, 2017; Vogley, 2019). For example, previous research has found that women with ADHD may have more difficulties with delayed gratification than men with ADHD, and that this can at least partly explain women with ADHD’s overall greater impairment (Doidge, 2017) Women with ADHD are also at enhanced risk for relationship problems (Tureau, 2004). Paradoxically, however, women with ADHD have higher educational attainment than men with ADHD (Velki & Vrdoljak, 2019). Given the apparent contradictory literature on the issue of gender, ADHD, and organizational issues, future research should consider the effect of gender in more detail.

With the caveat of gender still needing to be addressed in future research, the following indications for organizational skills’ interventions are suggested based on this study’s results:

1. **Focus on enhancing communication clarity, assiduity, and attentiveness.** This can be done through behavioural modeling and proper reinforcement procedures.
2. **Manage brain hyperactivity.** This may include methods to manage emotional dysregulation (i.e., the ability to calm oneself) and ways to “burn off” extra energy (e.g., sports and meditation).
3. **Introduce help at the moment of the performance itself.** That is, instead of a counselor being present to suggest overall organizational strategies, someone should be there to help the individual with ADHD implement the behaviour when it needs to be implemented.

## Limitations

The present study has several limitations. First, the study focused on biological sex rather than gender. While biological sex has a significant impact on a wide range of psychological disorders, obtaining additional information regarding the gender-identification of the participants could provide additional insights regarding the role of sex and gender in ADHD. Second, additional information regarding the diagnosis of ADHD, such as the time since the diagnosis or comorbidity, was not reported by the participants. Third, as mentioned previously, there is only minimal information regarding the psychometric properties of the DOSQ, especially regarding its convergent and discriminant validity. Additional research should specifically focus on examining the properties of this instrument.

## Conclusion

In conclusion, the current findings support that, while individuals with a diagnosis of ADHD may report lower organizational skills, they nevertheless attempt to use various strategies to remain organized. Our study provides a necessary insight into the organizational skills’ interventions needed for people with ADHD. It should be emphasized that people with ADHD oftentimes have many gifts and can be exceptionally creative as well as very productive in their areas of interest. To support and help individuals with ADHD and society benefit from these gifts, organizational skills are needed. This study therefore provides a crucial insight into organizational skills that will help those with ADHD reach their full potential.

##  Supplemental Information

10.7717/peerj.9844/supp-1 Supplemental Information 1 Raw data

## Additional Information and Declarations

### Data Availability

The following information was supplied regarding data availability: The raw data is available as a Supplemental File.
